# MASP-2 Is a Heparin-Binding Protease; Identification of Blocking Oligosaccharides

**DOI:** 10.3389/fimmu.2020.00732

**Published:** 2020-04-28

**Authors:** Ditmer T. Talsma, Felix Poppelaars, Wendy Dam, Anita H. Meter-Arkema, Romain R. Vivès, Peter Gál, Geert-Jan Boons, Pradeep Chopra, Annamaria Naggi, Marc A. Seelen, Stephan P. Berger, Mohamed R. Daha, Coen A. Stegeman, Jacob van den Born, the COMBAT Consortium

**Affiliations:** ^1^Department of Nephrology, University Medical Center Groningen, Groningen, Netherlands; ^2^Univ. Grenoble Alpes, CNRS, CEA, IBS, Grenoble, France; ^3^Institute of Enzymology, Research Centre for Natural Sciences, Budapest, Hungary; ^4^Department of Chemical Biology and Drug Discovery, Utrecht Institute for Pharmaceutical Sciences, and Bijvoet Center for Biomolecular Research, Utrecht University, Utrecht, Netherlands; ^5^Complex Carbohydrate Research Center, University of Georgia, Athens, GA, United States; ^6^Ronzoni Institute, Milan, Italy

**Keywords:** lectin pathway, MASP-2, tetrasaccharide, heparin, complement, glycosaminoglycans

## Abstract

It is well-known that heparin and other glycosaminoglycans (GAGs) inhibit complement activation. It is however not known whether fractionation and/or modification of GAGs might deliver pathway-specific inhibition of the complement system. Therefore, we evaluated a library of GAGs and their derivatives for their functional pathway specific complement inhibition, including the MASP-specific C4 deposition assay. Interaction of human MASP-2 with heparan sulfate/heparin was evaluated by surface plasmon resonance, ELISA and in renal tissue. *In vitro* pathway-specific complement assays showed that highly sulfated GAGs inhibited all three pathways of complement. Small heparin- and heparan sulfate-derived oligosaccharides were selective inhibitors of the lectin pathway (LP). These small oligosaccharides showed identical inhibition of the ficolin-3 mediated LP activation, failed to inhibit the binding of MBL to mannan, but inhibited C4 cleavage by MASPs. Hexa- and pentasulfated tetrasaccharides represent the smallest MASP inhibitors both in the functional LP assay as well in the MASP-mediated C4 assay. Surface plasmon resonance showed MASP-2 binding with heparin and heparan sulfate, revealing high Kon and Koff rates resulted in a Kd of ~2 μM and confirmed inhibition by heparin-derived tetrasaccharide. In renal tissue, MASP-2 partially colocalized with agrin and heparan sulfate, but not with activated C3, suggesting docking, storage, and potential inactivation of MASP-2 by heparan sulfate in basement membranes. Our data show that highly sulfated GAGs mediated inhibition of all three complement pathways, whereas short heparin- and heparan sulfate-derived oligosaccharides selectively blocked the lectin pathway via MASP-2 inhibition. Binding of MASP-2 to immobilized heparan sulfate/heparin and partial co-localization of agrin/heparan sulfate with MASP, but not C3b, might suggest that *in vivo* heparan sulfate proteoglycans act as a docking platform for MASP-2 and possibly prevent the lectin pathway from activation.

## Introduction

As a part of the innate immune system, complement consists of soluble, and cell bound proteins. The complement system is activated *via* three different pathways; the classical pathway (CP), lectin pathway (LP), and alternative pathway (AP). The CP is initiated by the binding of C1q to IgG or IgM and the LP by pattern recognition molecules binding to carbohydrates of pathogens or self-antigen. This leads to a conformational change and subsequent activation of the associated serine proteases C1r/C1s and MASP-1/MASP-2, respectively. These serine proteases cleave C2 and C4, forming the C4bC2a complex, a C3 convertase which deposits C3b initiating the amplification loop. The AP can be initiated either by auto activation of C3 eventually forming the C3 convertase C3bBb, or by *in situ* binding of AP stimulator properdin to the cell surface. Formation of the C5 convertase in the end leads to the generation of the C5b-9 membrane attack complex, resulting in cell lysis (1).

In the field of nephrology, complement has gained increased attention in recent years as studies have identified complement as a key player in multiple renal diseases. The classical pathway (CP) has been shown to play a major role in the auto-immune disease lupus erythematosus (2). In addition, lectin pathway (LP) components, either in plasma, or deposited within the kidney, have been correlated to disease progression following human kidney transplantation and hemodialysis, IgA nephropathy and diabetic nephropathy (3–6). Furthermore, it has been shown that mannan binding lectin (MBL) and collectin-11 recognize epitopes in I/R damaged kidneys and increase I/R induced damage (7, 8). Finally, the alternative pathway (AP) has been identified as a factor in the physiopathology of dense deposit disease, C3 glomerulopathy, atypical hemolytic uremic syndrome, and progression of proteinuric renal diseases (9–13). Therefore, complement-targeted therapies can potentially be of great use in a variety of renal diseases and conditions.

The *in vivo* inhibitory potential of heparin on the complement system has been known for ~25 years (14). Since then, numerous interactions have been described between glycosaminoglycans (GAGs) such as heparin, and complement components. In the lectin route of complement, anti-thrombin bound to heparin is a strong inhibitor of C4 cleavage by MASPs (15). Besides the lectin pathway, heparin can also block the classical pathway by directly inhibiting the C1q subunit of C1 or by potentiating the effect of C1-inhibitor (16–18). Studies by our group showed that the binding of both properdin, an alternative pathway initiator and stabilizer, and factor H, an alternative pathway inhibitor, to heparan sulfates (HS) on proximal tubular epithelial cells can be prevented by heparin and some other GAGs (12, 13). Combined, these studies indicate that GAGs have the potential to inhibit different components of the three pathways of the complement system.

Proteoglycans are glycoconjugates consisting of a core protein to which GAGs are covalently attached. Proteoglycans, such as the members of the syndecan and the glypican families, can be found on the cell membrane, others like versican, perlecan, and agrin are found in the extracellular matrix. Membrane proteoglycans function as highly abundant, relatively low affinity co-receptors for growth factors, chemokines, and adhesion molecules and modulate proliferation, migration, and adhesion events (19). Matrix-associated proteoglycans mostly function as storage depot for mediators, which can be released for paracrine functions upon tissue remodeling by proteases, sulfatases, and/or heparanase (20–22). GAGs consist of repetitive disaccharide units, which can be modified enzymatically in their length and sulfation pattern to alter their binding capacity and function. The effects on binding capacity and pharmacokinetics of GAG length modifications have been known for some time and have led to the introduction of low molecular weight heparins. Modifications of heparan sulfate (HS) sulfation are well-known from *in vivo* modifications of proteoglycan side chains upon stimuli like inflammation and fibrosis (23, 24). HS/Heparin can carry sulfate groups on the N position and/or on the 3-O, 6-O, and 2-O position of glucosamine and iduronic acid residues, respectively. Degree of sulfation is positively correlated to the electro-negativity of HS/Heparin and therefore to their binding capacity, making it an important factor in various cellular functions. It has for example been shown that the binding of heparin to anti-thrombin III depends on a specific pentasaccharide structure, in which 3-O sulfation is essential (25). Moreover, our group has shown that properdin and factor H require different GAG sulfation patterns for HS binding (13). These examples illustrate the important role of chain length and sulfation pattern of GAGs for specific interactions related to biological properties.

In this study, we aimed at identifying pathway-specific complement inhibiting GAGs from a library of natural and enzymatically and chemically modified and/or depolymerized GAGs. We show that small heparin- and HS-derived oligosaccharides are specific inhibitors of the LP of complement and that these sugars inhibit the LP *via* inhibition of the MASP-2 enzyme. By surface plasmon resonance we confirm MASP-2 to be a HS/heparin binding protein. Moreover, we provide some evidence that HS proteoglycans *in situ* might bind and eventually regulate MASP-2 *in vivo*.

## Materials and Methods

### Polysaccharides

Heparin from ovine intestinal mucosa, heparin from bovine lung, heparin (~2.5 sulfate groups/disacch) and HS from porcine intestinal mucosa (~1.0 sulfate groups/disacch), Chondroitin sulfate-A, -B, -and C, dextran T40, dextran sulfate, fucoidan, were purchased from Sigma (Sigma, Zwijndrecht, The Netherlands). Nadroparin (Fraxiparine^®)^ was purchased from Sanofi Winthrop (Maassluis, The Netherlands), Dalteparin (Fragmin®) was purchased from Pharmacia & Upjohn, and enoxaparin (Clexane®) was purchased from Rhone-Poulenc Rorer (Paris, France). HS isolated from bovine kidney (~0.8 sulfate groups/disacch) or from Engelbreth-Holm-Swarm sarcoma (~0.6 sulfate groups/disacch) were obtained from Seikagaku Corp (Tokyo, Japan). *Escherichia coli* capsular polysaccharide K5, with the same (GlcUA → GlcNAc)_*n*_ structure as the non-sulfated HS/heparin biosynthetic precursor polysaccharide (0.0 sulfate groups/disacch) (26); *O*-sulfated K5 and low molecular weight *O*-sulfated K5; were kindly provided by Dr. G. van Dedem (Diosynth, Oss, The Netherlands), as well as the heparin-derived octasaccharides (Org32100), hexasaccharides (Org 32101), and tetrasaccharides (Org 32102). HS from bovine intestine was kindly provided by Marco Maccarana (Department of Experimental Medical Science, Biomedical Center, University of Lund, Sweden) (27). HS from human aorta was isolated essentially as described by Iverius (~0.6 sulfate groups/disacch) (28). *N* + *O*-sulfated K5 was produced by *N*-deacetylation (hydrazinolysis), subsequent *N*-sulfation with sulfur trioxide-trimethylamine (29) of O-sulfated K5, followed by *N*-acetylation. Heparin and HS-derived oligosaccharides were prepared as previously described (30, 31) by partial heparinase I (Grampian enzymes, Orkney, UK) depolymerization of porcine mucosal heparin and exhaustive digestion of porcine mucosal HS with heparinase III (Grampian enzymes). Disaccharide analysis of SAGAG heparin tetrasaccharides was achieved by reverse-phase ion-pair high-performance liquid chromatography (RPIP-HPLC) as described before (32). Treatment of heparin by the 6-O-endosulfatase HSulf-2 was performed as described previously (33), for details on the disaccharide composition, see (34). Enoxaparin tetrasaccharides (Ron G11237) were kindly provided by dr. Annamaria Naggi (Ronzoni Institute, Milan, Italy) isolated and characterized by NMR spectroscopy as described before (35), N-desulfated, re-acetylated heparin and periodate-oxidized and reduced heparin were also provided by dr. Annamaria Naggi and were prepared and characterized as described before (36). Synthetic HS tetrasaccharides were kindly provided by prof. dr. G.J.P.H. Boons (Pharmacy, State University of Utrecht, The Netherlands) and were produced as described before (37).

### Wieslab Pathway-Specific Complement Assay

The Wieslab complement assay (WieLISA) kits were obtained from Euro Diagnostica, Malmö, Sweden. The WieLISA assay is a straightforward ELISA-based format for the evaluation of the three pathways of complement activation. The assays are based on specific coatings for each pathway in combination with specific buffer systems and measure the deposition of the terminal C5b-9 MAC complex. The measurement of the lectin pathway is either done by using mannan coated plates, allowing binding of the MBL-MASP complex from serum (in the standard WieLISA assay), or by using acetylated BSA coated plated, allowing binding of ficolin-3-MASP complexes from serum (in the ficolin-3 LP assay). The assays have been described by Seelen et al. (38). Positive control serum delivered with the kits was used as serum source for all measurements. GAGs were diluted in route specific buffer delivered with the kit. Final GAG concentration was 100 μg/ml in the CP and LP assay and due to a higher incubated serum concentration 200 μg/ml in the AP assay. Serum was added to the diluted GAGs right before the plates were incubated at 37°C for 60 min. Except for the serum (+/– GAGs) incubation, the kit protocol was followed. Data were expressed as % inhibition compared to the positive control. Dose dependent assays with variable amounts of GAGs were performed as described above. Data was presented as representative experiments.

### MBL Binding Assay

To evaluate whether GAGs could inhibit the mannan—MBL interaction, Maxisorp immunoassay plates were incubated overnight with 100 μg/ml mannan (Sigma, Zwijndrecht, The Netherlands) diluted in 0,1 M NaCO_3_, pH 9,6. After washing three times, plates were blocked with 1% BSA in PBS for 1 h. Thereafter, pooled serum diluted 1:50 in GVB++ buffer, described by Roos et al. (39), were pre-incubated with a concentration range of GAGs, for 15 min at room temperature. GVB++ buffer is Veronal-buffered saline (1,8 mM Na-5,5-diethylbarbital, 0,2 mM 5,5-diethylbarbituric acid, 145 mM NaCl) containing 0,5 mM MgCl2, 2 mM CaCl2, 0,05% Tween-20 and 0,1% gelatin, pH 7.5. The pre-incubated mixture was then incubated for 60 min on the mannan coated plates at 37°C. In the dose dependent binding of MBL to mannan, no GAGs were added. Bound MBL was detected with a DIG-labeled mouse anti-human MBL antibody 1:1,000 (mAb 3e7 from Hycult Biotech, Uden, the Netherlands) and a Sheep anti-DIG HRP labeled conjugate 1:8,000 (Roche Diagnostics, Mannheim, Germany). The assay was developed using tetramethylbenzidine (TMB) (Sigma, Zwijndrecht, The Netherlands) and the reaction was stopped with 1 M H2SO4. Absorbance was measured at 450 nm in a microplate reader. Data was expressed as % inhibition compared to non-inhibited control. Data was presented as representative experiment.

### C4 Inhibition Assays

To evaluate whether GAGs inhibit the C4 cleavage (by C4d deposition) by MASPs, we performed a C4 cleaving assay, as first described by Petersen and colleagues (40). Maxisorp immunoassay plates were incubated overnight with 100 μg/ml mannan (Sigma, Zwijndrecht, The Netherlands) diluted in 0,1 M NaCO_3_, pH 9,6. Thereafter, plates were blocked for 1 h using 10 mM Tris-HCl, 140 mM NaCl, 0,1% BSA, pH 7,4. Next, pooled serum diluted 1:100 in 20 mM Tris-HCl, 10 mM CaCl_2_, 1 M NaCl, 0,05% Triton X-100, 0,1% BSA, pH 7,4 was incubated overnight at 4°C, allowing the MBL/MASP complex to bind to mannan, but prevents MASP activation and subsequent complement activation. Incubation of serum in 1 M NaCl was done to prevent classical pathway activation through anti-mannan IgG3 antibodies, prevalent in the majority of the healthy population. High ionic buffers prevent the binding of C1q to immune complexes and disrupt the C1 complex, whereas the carbohydrate-binding activity of MBL and the integrity of the MBL complex are maintained under hypertonic conditions. After washing, MBL/MASP coated plates were pre-incubated with 50 μl GAG at twice the final concentration in 10 mM Tris-HCl, 140 mM NaCl, 5 mM CaCl_2_, 0,05% Tween, 0,1% BSA, pH 7,4 for 30 min at 37°C. Non-inhibited control wells were incubated with buffer only. Without washing, 50 μl of 5 μg/ml purified C4 (Hycult Biotech, Uden, the Netherlands) diluted in 10 mM Tris-HCl, 140 mM NaCl, 5 mM CaCl_2_, 0,05% Tween, 0,1% BSA, pH 7,4 was added to the pre-incubated GAGs and incubated for 1 h at 37°C. After washing, C4d deposition was detected using a DIG labeled mouse anti-human C4 antibody, diluted 1:4,000, followed by incubation with a Sheep anti-DIG HRP labeled antibody (Roche Diagnostics, Mannheim, Germany, dilution 1:8,000). The assay was developed using TMB (Sigma, Zwijndrecht, The Netherlands) and the reaction was stopped with 1 M H_2_SO_4_. Absorbance was measured at 450 nm in a microplate reader. Data was expressed as % inhibition compared to non-inhibited control. Experiments were independently reproduced in triplicate.

### Heparin-MBL/MASP Interaction ELISA

In three independent experiments, we tested the binding of serum MASP-2 and recombinant MASP2 to heparin. To this end, we coated Maxisorp immunoassay plates overnight with 5 μg/ml heparin-albumin or 5 μg/ml albumin diluted in PBS. Heparin-albumin was from Sigma-Aldrich (Saint Louis, MO, USA). According to the data sheet, this artificial proteoglycan contained 4.8 moles heparin per mole albumin, protein content is about 55%. After washing, plates were blocked using 1% BSA in PBS for 1 h. Thereafter, recombinant MASP2 or pooled human serum diluted in GVB++ buffer was incubated for 2 h at 4°C. Binding of MASP-2 was detected by incubating rat anti-human MASP-2 (Hycult Biotech, Uden, the Netherlands) diluted at 1:200 in PBS, 1% BSA and 0,05% Tween. Rabbit anti-Rat HRP labeled (DAKO, Glostrup, Denmark) diluted 1:500 in PBS, 1% BSA and 0,05% Tween was incubated for 1 h. The assay was developed using TMB (Sigma, Zwijndrecht, The Netherlands), the reaction was stopped with 1 M H_2_SO_4_. Absorbance was measured at 450 nm in a microplate reader.

### Surface Plasmon Resonance Analysis

To define the binding affinity of MASP-2 to heparin derivatives surface plasmon resonance (SPR) experiments were performed using recombinant human MASP-2 protein containing the catalytic fragment (CCP1-CCP2-SP), which was prepared as described earlier (41). All experiments were performed on a BIAcore T200 (GE healthcare), using as previously described standard procedures and GAG biotinylation techniques (40). Briefly, reducing-end biotinylated 6 and 15 kDa heparin, and HS (porcine mucosa) were captured on three streptavidin-activated flowcells of a S-CM4 sensorchip (41.2 and 44.1 RU for 6 and 15 kDa heparin respectively, and 61.9 RU for HS, a fourth one being used as negative control surface. Using HBS-P+ running buffer (10 mM HEPES, 150 mM NaCl, 0.05% surfactant P20, pH 7.4) at a flow rate of 20 μl/min, series of 0–3,000 nM of MASP-2 were injected over the heparin, HS and negative control surfaces, followed by a 3 min washing step with HBS-P+ buffer to allow dissociation of the complexes formed. At the end of each cycle, surfaces were regenerated with a 2.5 min injection of 2M NaCl. Sensorgram shown correspond to on-line subtraction of the negative control to the heparin surface signal. Kds were determined using the BIAcore T200 evaluation (version 3.1) software, by steady state analysis (1:1 binding model). For this, resonance values (Req) were taken within the equilibrium phase (just before the end of injection) and plotted against MASP-2 concentration. Calculated χ2 values provided quality control of experimental data fitting. Competition assays were performed similarly, by injecting 200 nM of MASP-2 pre-incubated with GAGs (30 μg/ml) over the heparin and negative control surfaces. Tested GAGs included a heparin tetrasaccharide (ΔHexA2S-GlCNS6S-IdoA2S-GlcNS6S) and chondroitin sulfate A, B and C, the 15 kDa heparin being used as a positive control (also 30 μg/ml, a 10-fold excess compared to the concentration used to load the sensor chip). Noteworthy, this experiments was conducted using fixed tetrasaccharide and heparin mass concentrations to reflect the fact that a 15 kDa heparin chain comprises multiple highly sulfated tetrasaccharide (composition of 15 kDa Heparin is provided in Seffouh et al. (42). All experiments were independently replicated three times.

### Immunohistochemistry

Four μm frozen kidney sections of five human donor kidneys not suitable for transplantation for anatomical reasons were used for immunofluorescent stainings. Sections were double stained for MASP-2 in combination with the basement membrane HS proteoglycan agrin, or in combination with HS (clone 10E4 recognizing mixed N-sulfated/N-acetylated sequences) or triple stained with HS and activated complement factor C3 (recognizing a neoepitope on C3b, iC3b, and C3c after cleavage of C3 by C3-convertase). Details of the immunofluorescence procedures and the antibodies used are given in Table 1. Photomicrographs were taken at 630x magnification with confocal microscope (Leica SP8, Leica microsystems BV, Rijswijk, the Netherlands) at the Imaging and Microscopy Center of the University Medical Center Groningen.

**Table 1:** Details on immunofluorescence stainings on human cryosections.

**Procedure**	**MASP-2**	**Agrin**	**Heparan sulfate**	**Activated C3**
Fixation	Icecold 100% aceton for 10 min	Icecold 100% aceton for 10 min	Icecold 100% aceton for 10 min	Icecold 100% aceton for 10 min
Peroxidase inactivation	0.03% H_2_O_2_ in PBS for 30 min in the dark	–	–	–
Blocking a-specific background	1% BSA in PBS for 15 min	1% BSA in PBS for 15 min	1% BSA in PBS for 15 min	1% BSA in PBS for 15 min
Blocking endogenous biotin	–	–	Avidin/Biotin blocking kit (SP-2001; Vector Laboratories; Burlingame CA, USA)	–
Primary antibody	Rat mAb anti-human MASP-2, clone 8B5, 1:100 (Hycult, Uden, The Netherlands)	Mouse mAb JM-72 anti-human agrin, 1:750 (43)	Biotinylated mouse mAb anti-heparan sulfate, clone 10E4, 1:50 (Amsbio, Abingdon, UK)	Mouse IgG2a mouse mAb anti-neoepitope on C3b, iC3b and C3c, clone bH6, 1:200 (Hycult)
Secondary antibody	Rabbit anti-rat IgG-HRP, 1:200 + 5% normal human serum for (Dako, Glostrup, Denmark)	Donkey anti-Mouse IgG-Alexa 488, 1:250 (Life Technologies, Carlsbad CA, USA)	–	Donkey anti-mouse IgG-Alexa647, 1:250 (Life Technologies, Carlsbad CA, USA)
Tertiary antibody	Goat anti-rabbit IgG-HRP, 1:200 + 5% normal human serum (Dako)	–	–	–
Amplification HRP signal	TSA Tyramide-TRITC, 1:50 for 10 min (PerkinElmer, Waltham MA, USA)	–	–	–
Detection biotinylated antibody	–	–	Streptavidin-FITC, 1:300 (Invitrogen, Waltham MA, USA)	–
Nuclear staining	Dapi for 10 min	Dapi for 10 min	Dapi for 10 min	Dapi for 10 min
Embedment	Citifluor (Haffield PA, USA)	Citifluor (Haffield PA, USA)	Citifluor (Haffield PA, USA)	Citifluor (Haffield PA, USA)

*Stainings are performed as MASP2/agrin double staining, as MASP2/HS double staining, and as MASP2/HS/actC3 triple staining*.

*All antibody incubations were done for 30 min at room temperature in PBS + 1% BSA. Sections were washed with PBS in between the incubation steps. Double- and triple stainings were designed in such a way that all cross-reactions among the stainings were avoided*.

## Results

### Small Heparan Sulfate- and Heparin-Derived Oligosaccharides Specifically Inhibit the Lectin Pathway, While Longer Highly Sulfated GAGs Block All Three Routes of Complement

A library of naturally, chemically, or enzymatically modified and synthetic GAGs, as well as size-defined HS/heparin depolymerization products were tested for their complement inhibiting potential in the WieLISA, which allows separate evaluation of the three pathways of the complement system. From the results (Table 2), it can be seen that unfractionated heparins inhibited all three complement pathways. Heparin from different sources e.g., porcine intestinal mucosa and bovine lung showed strong inhibitory potential for all pathways.

**Table 2:** Complement inhibition by heparin(oids), (derivatives of) K5 polysaccharide, (derivatives of) heparan sulfates, and some other glycosaminoglycans.

**Glycosaminoglycans**	**Classical pathway**	**Lectin pathway**	**Alternative pathway**	**Repeats**
**HEPARINS AND HEPARIN DERIVATIVES**				
Porcine intestinal mucosa	73 (12)	97 (1)	94 (5)	2
Ovine intestinal mucosa	83	97	96	1
Bovine lung	66	90	74	1
SULF-2 treated heparin	54	96	48	1
N-desulfated, re-acetylated heparin	6	52	42	1
Periodate-oxidized and reduced heparin	60	91	97	1
LMW-heparin (Fragmin) Mw: 6000	73	98	98	1
LMW-heparin (Fraxiparin) Mw: 4500	48	95	92	1
LMW-heparin (Enoxaparin) Mw: 4500	65	97	95	1
LMW N-desulfated, reacetylated heparin	8	46	44	1
Heparin-derived 18-mer	13	71	20	1
Heparin-derived 16-mer	27	90	25	1
Heparin-derived 14-mer	26	92	35	1
Heparin-derived 12-mer	26	94	38	1
Heparin-derived 10-mer	14	87	21	1
Heparin-derived 8-mer	20	93	26	1
Heparin-derived 6-mer	12	93	0	1
Heparin-derived 4-mer	0	74	0	1
***E. coli*** **K5-DERIVED POLYSACCHARIDES**				
Native K5	0	1	3	1
O-sulfated K5	100 (0)	88 (4)	92 (3)	3
N-+ O-sulfated K5	100	93	97	1
O-sulfated K5 hexasaccharides	0	2	0	1
**GLYCOSAMINOGLYCANS**				
Chondroitin sulfate A	3 (4)	6 (7)	8 (9)2	3
Chondroitin sulfate C	0 (0)	0 (0)	0 (0)	2
Chondroitin sulfate B (Dermatan sulfate)	5 (7)	39 (5)	10 (10)	3
Sulodexide	52	93	73	1
**POLYSACCHARIDES**				
Dextran T40	4	8	17	1
Dextran sulfate	100	99	100	1
Fucoidan	98	78	79	1
**HEPARAN SULFATE AND HEPARAN SULFATE DERIVATIVES**				
HS human aorta	5	23	7	1
HS EHS mouse sarcoma	17	31	22	1
HS bovine intestine	21	76	39	1
HS bovine kidney	2 (1)	73 (0)	18 (18)	2
HS porcine mucosa	9	95	26	1
Heparan sulfate derived 18-mer	5	91	25	1
Heparan sulfate derived 16-mer	11	92	32	1
Heparan sulfate derived 14-mer	4	92	18	1
Heparan sulfate derived 12-mer	0	93	3	1
Heparan sulfate derived 10-mer	0	87	0	1
Heparan sulfate derived 8-mer	0	74	0	1
Heparan sulfate derived 4-mer	0	0	0	1

*A library of GAG-derived polysaccharides was tested in the WieLISA for their complement inhibiting potential. Values are expressed as percentage inhibition compared to control values without inhibitor as mean ± SD or from a single experiment. GAGs were added in a concentration of 100 ug/ml in the CP and LP assay and due to a higher serum concentration at 200 ug/ml in the AP assay*.

Selective desulfation of heparin, either 6-O desulfation by SULF2 (80% reduction of the 6-O content in NS2S6S disaccharides), or by chemical N-desulfation followed by reacetylation, resulted in some loss of complement inhibitory potential, mostly in the classical and alternative route.

Periodate-oxidized and reduced heparin is a non-anticoagulant heparin derivate in which the structure of the antithrombin binding site was modified, and the results in Table 2 show that despite losing the ability to interact with antithrombin III, it remained able to inhibit all complement pathways. LMW-heparins like fragmin, fraxiparin and enoxaparin are widely used in the clinic as anticoagulants and showed strong potential to inhibit the three complement pathways. N-desulfation followed by reacetylation of LMW heparin reduced the ability to inhibit all complement pathways, mostly the classical pathway. Size fractionation products of limited heparinase I treated heparin showed that smaller heparin fragments retain their ability to inhibit the LP, but were no longer able to inhibit the CP and AP, indicating that GAG chain length is a major determinant for CP and AP inhibition. The smallest heparin fragment tested, i.e., tetrasaccharides (4-mer), were found to be completely selective for inhibition of the LP.

*Escherichia coli*-derived K5 polysaccharides share their polysaccharide backbone with heparin except that K5 polysaccharides carry exclusively GlcA uronic acid epimers, while heparin features >80% IdoA C5 uronic epimers. Native K5 does not contain any sulfate groups and did not inhibit the complement system. O-sulfated and N + O-sulfated K5 both showed equally strong inhibitory capacity for all three complement pathways, indicating that neither GlcA to IdoA conversion nor N-sulfation (next to O-sulfation) is crucial for complement inhibition, when the density of O-sulfates is high enough (~1,5 O-sulfates/disacch in O-sulfated K5). O-sulfated K5 hexasaccharide did not inhibit the complement unlike heparin hexasaccharides (see above). Whether this is due to different positioning of the O-sulfate groups is unknown, but suggestive, which in regular heparin is mostly IdoA2S-GlcNs6S, and in chemically O-sulfated K5 mostly at C2 and C3 of GlcA along with N-sulfation of Glc units.

We also tested GAGs and polysaccharides with a different backbone structure than heparin. These results indicate that the chemical nature of the monosaccharides and their bonds between the monosaccharide units is of lesser importance compared to the degree of sulfation when looking at complement inhibitory potential. Highly sulfated polysaccharides like fucoidan and dextran sulfate and to a lesser extent sulodexide [mixture of heparin (80%) and dermatan sulfate (20%)] showed inhibition of all complement pathways. Chondroitin sulfate B (dermatan sulfate) with an intermediate amount of sulfates and high content of iduronic acid showed some inhibition of the LP, but not of the CP and AP, while chondroitin sulfates -A and -C and the non-sulfated dextran T40 didn't inhibit the three complement routes.

**Figure 1:**
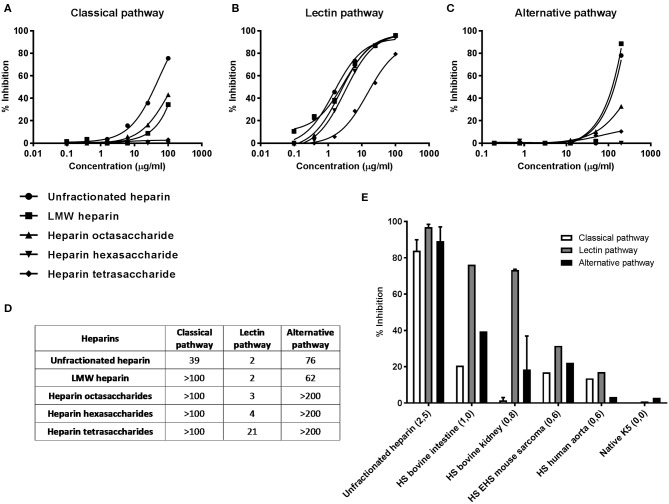
Effect of GAG length and sulfation on complement inhibition. Complement inhibitory potential of selected heparin derivatives was evaluated in a dose dependent fashion. The concentration-response curves show that all heparin derivatives tested are strong LP inhibitors **(B)**, while the CP and AP are only inhibited by heparin and LMW heparin in concentrations of ≥50 μg/ml **(A,C)**. Heparin hexa-and tetrasaccharides are fully specific LP inhibitors **(A–C)**. **(D)** IC50 values (μg/ml) of the dose dependent inhibition assays **(A–C)** of the heparin fragments tested. **(E)** Illustration of the role of sulfation on complement inhibition. The average amount of sulfate groups per disaccharide is between brackets. Heparin shows inhibitory potential for all pathways. Reducing the number of sulfate groups per disaccharide results predominantly in a reduced potential to inhibit the CP and AP while the inhibitory potential for the LP is initially preserved. GAGs were tested at 100 (CP and LP) or 200 μg/ml (AP). Data shown are the result of a single experiment, after careful optimization of all conditions before. Heparin and HS from bovine kidney **(E)** as mean +/– SEM of two independent experiments.

HS have a slightly different disaccharide composition compared to heparin and are less and more variably sulfated (25, 44). HS from different sources (e.g., bovine kidney and porcine mucosa) showed in general substantial LP inhibition without inhibiting the CP and AP. Smaller HS fragments were found to be more specific inhibitors of the LP. 12-mer, 10-mer and 8-mer HS fragments showed complete specificity for the LP and did not show any inhibition of the CP and AP (Figures 1A,C,D). The HS-derived 4-mer did not show any inhibitory capacity for any pathway (HS-derived 6-mer was not available) in contrast to heparin-derived tetrasaccharide, probably because of a lower degree of sulfation (Table 2).

To test the effect of GAG length on the inhibitory potential of the complement system, 5 heparin fragments ranging from unfractionated heparin to heparin-derived tetrasaccharides were tested in the WieLISA in a dose dependent fashion. As expected, unfractionated heparin showed the strongest inhibition in all pathways (Figures 1A–C). Interestingly, the LP was inhibited most potently by unfractionated heparin with an IC50 value of 2 μg/ml in 100-times diluted serum, in contrast to the CP (IC50: 39 μg/ml) and the AP (IC50: 76 μg/ml) (Figure 1D). Heparin octasaccharides and smaller heparin fragments become, up to the concentrations tested, specific LP inhibitors; IC50: octasaccharides: 3 μg/ml, hexasaccharides: 4 μg/ml and tetrasaccharides: 21 μg/ml (Figure 1D). These results indicate that a certain heparin chain length is required for inhibition of the CP and AP, while heparin fragments down to 4 saccharides in length can inhibit the LP.

To illustrate the effect of heparin/HS sulfation on the inhibitory capacity of complement, we selected 6 heparin/HS/K5 preparations with different sulfation degrees from Table 1 and displayed them in Figure 1E. Heparin with >2,5 sulfate groups per disaccharide showed, as observed before, strong inhibitory potential for all complement pathways. A reduced number of sulfate groups to 1,0 or 0,8 per disaccharide, attenuated predominantly the ability to inhibit the CP and AP. Further lowering the sulfate content resulted in a reduced inhibition of all complement pathways (Figure 1E).

Altogether, all three complement pathways can be blocked by highly sulfated polysaccharides such as heparin, fucoidan, dextran sulfate and O-sulfated K5 and to a lesser extent by some HS preparations and dermatan sulfate. Specific complement inhibition of the LP is achieved by some HS preparations and small heparin- and HS-derived oligosaccharides.

### Heparin Oligosaccharides Inhibit the Proteolytic Activity of MASPs and Thereby Reduce C4d Deposition

**Figure 2:**
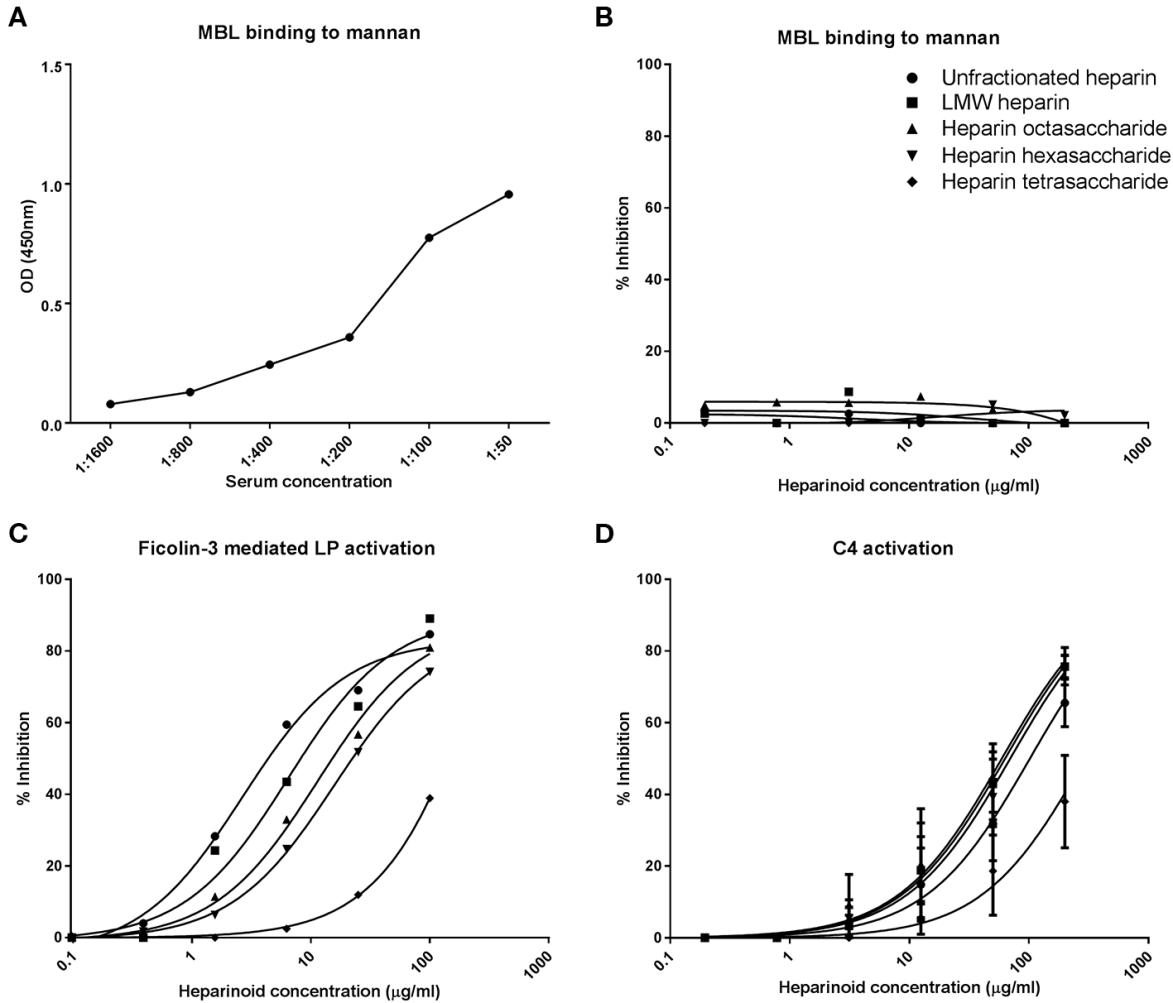
Heparin-derived oligosaccharides inhibit the lectin pathway of complement *via* inhibition of C4d deposition. To determine which LP component is inhibited by heparin derivatives MBL and MASP inhibition were tested. Representative experiments show that MBL binds dose dependent to mannan **(A)**. Heparin derivatives did not inhibit the binding of MBL to mannan **(B)**. The selected heparin fragments did however show inhibition of the LP when the LP was initiated by ficolin-3 binding **(C)**. Heparin fragments did also show an inhibitory effect in a C4d deposition assay, as a measure for MASP-2 activity **(D)**. Data is expressed as a representative measurement **(A–C)** or as mean ± SEM of three independent experiments **(D)**.

The previous results showed that HS/heparin-derived oligosaccharides can specifically inhibit the LP of complement. The LP differs from the CP only in the pattern recognition molecule: MBL (in the WieLISA) vs. C1q, and the serine proteases, MASP-1 and−2 vs. C1r and C1s. To pinpoint whether the small heparin oligosaccharides interfere with MBL or MASPs, the inhibitory effect of heparin (fragments) on the MBL-mannan interaction was tested. Serum was co-incubated with or without the heparin (fragments) on a mannan coated plate and MBL binding to mannan was used as a read out. MBL binding to mannan in the absence of inhibitors showed a dose dependent binding, a serum concentration of 1:50 was used for the inhibition experiments (OD: 0.96) (Figure 2A). The results revealed that none of the selected heparin preparations, which all inhibit the LP in the WieLISA, could inhibit the MBL binding to mannan in any of the concentrations tested (Figure 2B). To strengthen the conclusion that heparin did not interfere with the MBL-mannan interaction, heparin (fragments) were tested in a ficolin-3 mediated LP activity assay. This assay measures LP activity with ficolin-3 as pattern recognition molecule for immobilized acetylated BSA instead of MBL with immobilized mannan. In ficolin-3 mediated LP activation cleaving of C4 and C2 is, like in the MBL mediated route, dependent on MASP activity (45). The heparin (fragments) showed a dose dependent inhibitory pattern in the ficolin-3 mediated LP assay identical to the MBL mediated WieLISA (Figure 2C vs. Figure 1B). Unfractionated heparin showed the strongest inhibitory effect with an IC50 of 3 μg/ml. Decreasing the GAG length resulted in reduced inhibitory potential for LMW heparin, octasaccharides, hexasaccharides, and tetrasaccharides heparin (IC50: 7, 11, 16, and 342 μg/ml, respectively). Since the LP and CP differ only in their pattern recognition molecules and their serine proteases and we showed that the heparin (oligoaccharides) are unable to inhibit the binding of pattern recognition molecules to pathogen associated molecular patterns, we suggest, based on these data, that the LP is inhibited by heparin (oligosaccharides) via the serine proteases, the MASP enzymes.

To prove this assumpsion, we next used an assay to measure the C4 cleavage potential (by measuring C4d deposition) of the MASPs. Since C4 is completely cleaved by MASP-2 and not by MASP-1, this is predominantly a MASP-2 assay (46). Interestingly, unfractionated heparin showed a relatively mild inhibitory potential (IC50: 102 μg/ml) compared to the LMW-heparin enoxaparin, heparin oligosaccharide and heparin hexasaccharide (IC50: 63, 59, and 70 μg/ml, respectively). The weakest inhibitory effect was shown by the heparin tetrasaccharide with an IC50 of 296 μg/ml (Figure 2D). This assay revealed that heparin oligosaccharides inhibit the LP at the level of the MASP enzymes, most likely MASP-2, in a dose dependent manner.

### Recombinant Human MASP-2 Shows Similar Binding Affinity for Short and Long HS GAGs

**Figure 3:**
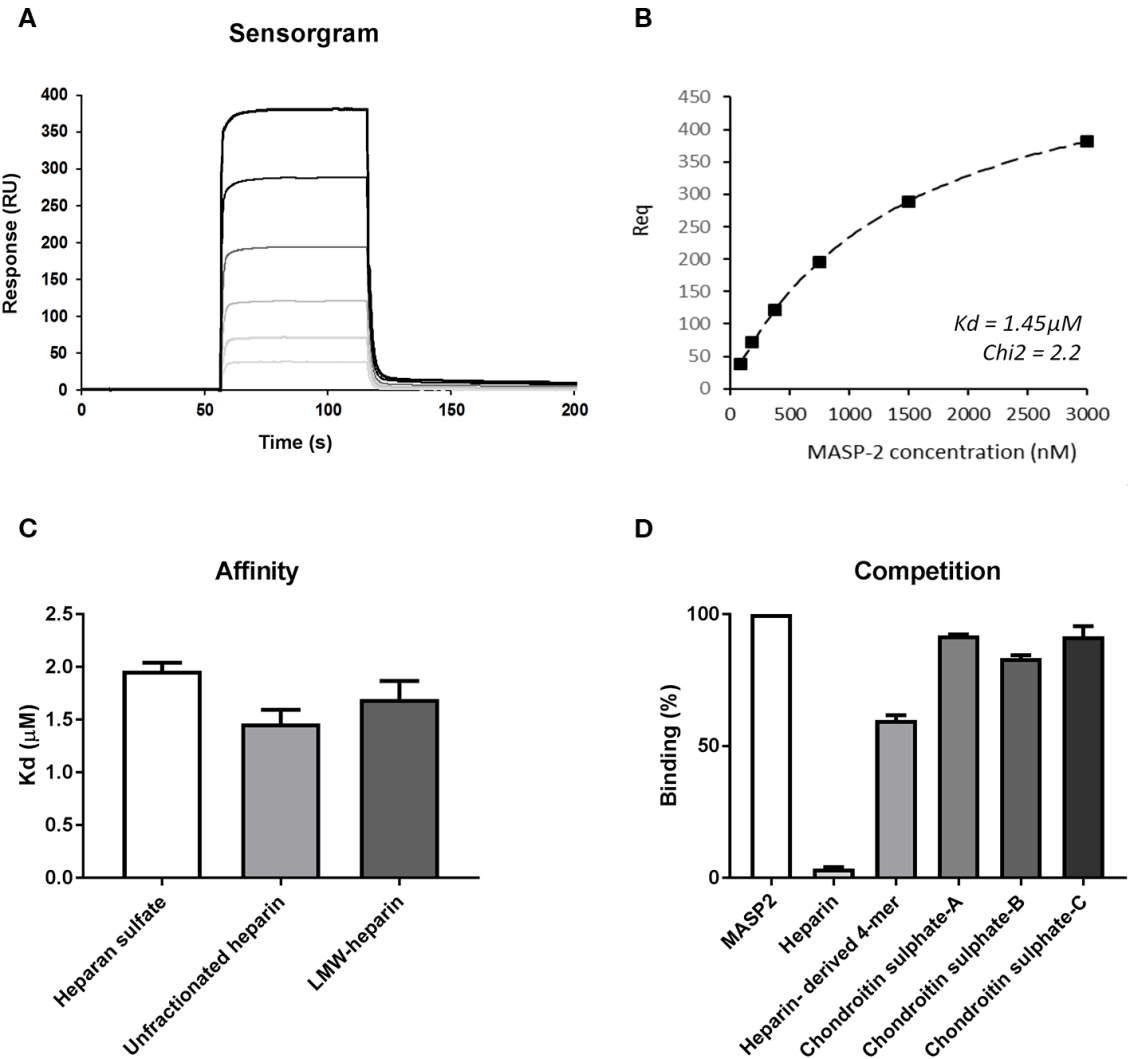
The recombinant catalytic CCP1-CCP2-SP domain of MASP-2 shows equal affinity for heparan sulfate, small, and larger heparin preparations. **(A)** Surface plasmon resonance sensorgram of binding of a (from clear to dark lines) 0–3,000 nM concentration range of recombinant MASP-2 to 15 kDa heparin, showing rapid on and off rates, indicating a transient interaction. Binding of MASP-2 to HS and 6 kDa heparin produced similar graphs (Supplementary Figure 1). **(B)** Representative steady-state analysis and fit of the experimental data for the binding of MASP-2 to 15 kDa heparin. **(C)** Affinity calculations reveal micro molar affinity for MASP-2 binding to HS, 6 kDa, and 15 kDa heparin. **(D)** Competition experiments using a tetrasaccharide (4-mer) heparin fragment results in a 40% reduction of the binding of MASP-2 to immobilized 15 kDa heparin, while chondroitin sulfates fail to inhibit the MASP-2/heparin interaction. Experiments were expressed as mean ± SEM from three independent experiments.

To further strengthen the hypothesis that heparin/HS fragments predominantly inhibit MASP-2 we performed SPR experiments to evaluate the binding kinetics between catalytically active recombinant human MASP-2 fragment (CCP1-CCP2-SP) and different heparin/HS preparations. Figure 3A shows the sensorgram of recMASP-2 binding to an immobilized 15 kDa heparin fragment. Immobilized HS (porcine mucosa) and 6 kDa LMW-heparin showed similar sensorgrams (Supplementary Figure 1). Interestingly the on and off rates were very fast indicating a transient interaction, which could indicate moderate affinity. In Figure 3B we show the fit of the experimental data for the binding of MASP-2 to 15 kDa heparin upon representative steady-state analysis. Calculations of the affinity indeed show Kd values between 1.5 and 2.0 μM for the heparins and HS tested (Figure 3C). The observation that MASP-2 showed similar affinity for the 6 kDa heparin and the 15 kDa heparin and HS (usually between 12 and 20 kDa) indicates that MASP-2 recognizes a rather short and comparable motif on heparin/HS. Using the 15 kDa heparin as a scaffold for MASP-2, competition assays were performed using unfractionated heparin, heparin-derived tetrasaccharide (identical composition as Org 32102, namely ΔHexA2S-GlCNS6S-IdoA2S-GlCNS6S) and chondroitin A, B, and C as competitors. Results show that the heparin-derived tetrasaccharide reduced the binding of MASP-2 to 15 kDa heparin by ±40%. Although further experiments (including direct MASP-2/tetrasaccharide binding analysis) would be needed to ascertain that a tetrasaccharide is the actual minimal size required for MASP-2 binding, these data again suggest that MASP-2 is recognized by a short heparin/HS motif (Figure 3D).

### Sulfation Pattern Determines the MASP-2-Mediated Lectin Pathway Inhibitory Potential of Tetrasaccharides

**Figure 4:**
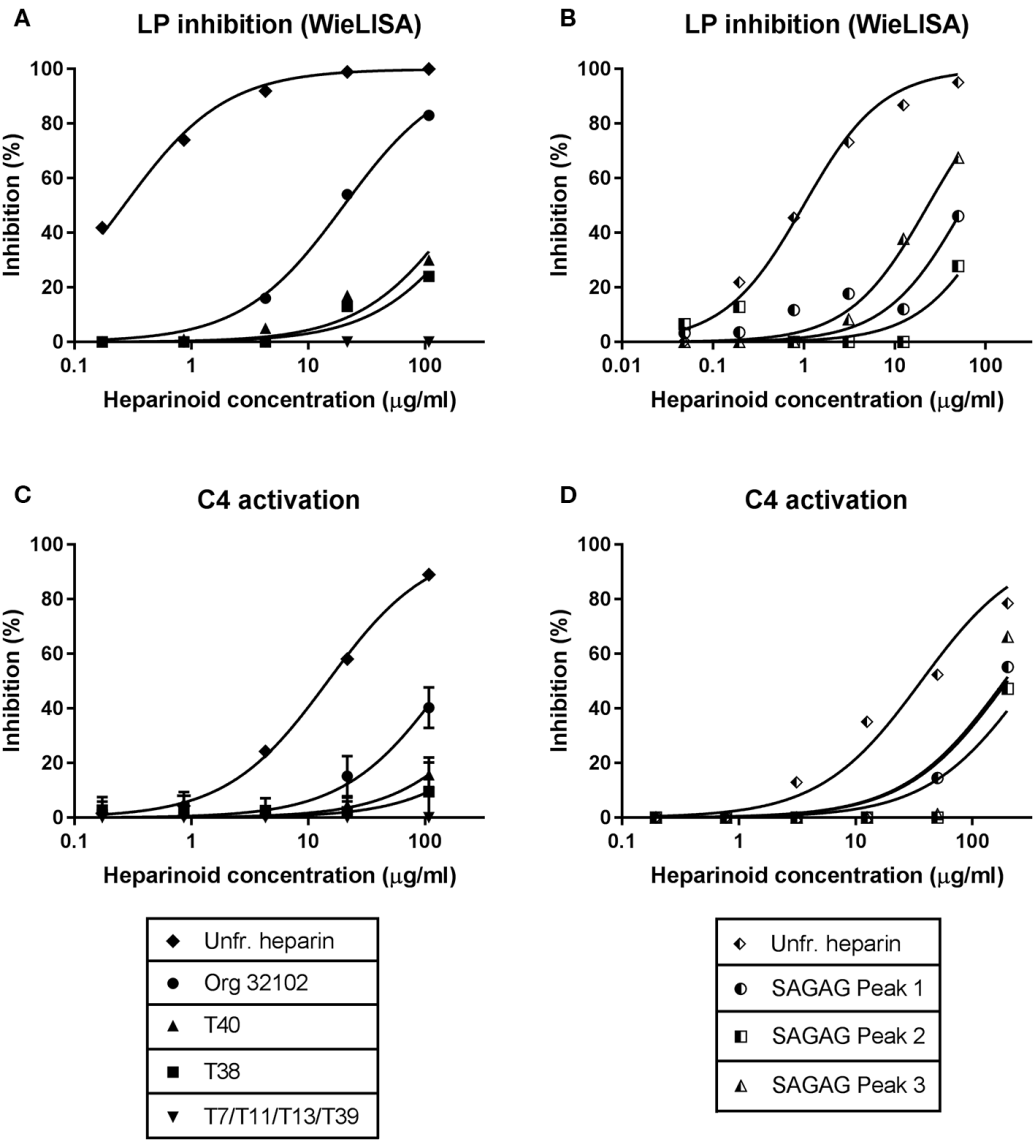
Inhibition of the lectin pathway by tetrasaccharides requires at least pentasulfation. Using synthetic tetrasaccharides **(A,C)** and purified tetrasaccharides **(B,D)**, we determined that LP inhibition requires at least a pentasulfated tetrasaccharide. All penta- and hexa-sulfated oligosaccharides showed inhibition in both the LP WieLISA and the C4 activation test. Removing both IdoA2S sulfate groups or both N-sulfate groups resulted in vanishing of the inhibitory potential. Displacement of an iduronic acid by an unsaturated uronic or hexuronic acid results in an improvement of inhibitory potential in both assays. For data on the sulfation degree of the tetrasaccharides see Table 3. As a reference the non-synthetic heparin tetrasaccharide 32102 and unfractionated heparin, used in the former experiments, was added to experiment **(A,C)**. Data is expressed as representative measurement **(A,B,D)** or as the mean ± SEM of three independent experiments **(C)**.

To determine the importance of sulfate group positioning within tetrasaccharides for the inhibition of the LP, various heparin-derived tetrasaccharides obtained after limited heparinase I digestion as well as a set of synthetic tetrasaccharides were tested in the WieLISA and the C4 activation assay (37). As a reference the heparin tetrasaccharide Org 32102 (>90% ΔUA2S-GlcNS6S-IdoA2S-GlcNS6S as evaluated by NMR) and unfractionated heparin used in the experiments above were added. The tetrasaccharides were tested in both the WieLISA and MASP-2-mediated C4 deposition assay. None of the tetrasaccharides affected the CP and the AP (data not shown). As shown before, results in the LP WieLISA and the MASP-mediated C4 deposition assay showed comparable results (Table 3 and Figure 4). Strongest inhibition in both assays was obtained by the fully hexasulfated ΔUA2S-GlcNS6S-IdoA2S-GlcNS6S (Org 32102) tetrasaccharide. The hexasulfated preparations with the ΔUA2S at the non-reducing end (by heparinase I elimination reaction), like Org32102 and SAGAG peak 3, showed better inhibitory capacity compared to the synthetic variant, tetrasaccharide T40. Pentasulfated tetrasaccharides also showed inhibitory capacity in both assays, although to a lesser extent compared to the hexasulfated tetrasaccharide. Thus, SAGAG Peak 1 and 2 (having 5 sulfate groups) vs. SAGAG peak 3 (six sulfate groups), and synthetic tetrasaccharide T38 (having 5 sulfate groups) vs. T40 (having 6 sulfate groups). The data indicate that one of the 2-O or 6-O sulfates within the tetrasaccharide can be missed, leaving a pentasulfated HS/heparin tetrasaccharide being the minimal MASP-2 binding sequence in HS and heparin. Further reduction in sulfation to four or less sulfate groups/tetrasaccharide led to loss of all inhibitory potential in both the LP Wielisa and the MASP-2 C4 deposition assay (Table 3). Not tested in these experiments are the influence of 3-O sulfation and the eventual effect of GlcNAc or ${\text{GlcNH}}_{3}^{+}$ modifications.

**Table 3:** Inhibitory capacity of heparin-derived and synthetic tetrasaccharides in the Lectin Pathway WieLISA and MASP-mediated C4 deposition assay.

**Tetrasaccharide**	**Structure**	**Inhibition (%) in WieLISA**	**Repeats**	**Inhibition (%) in C4 assay**	**Repeats**
**BY HEPARINASE I TREATMENT OF (LMW-) HEPARIN**					
Org 32102	ΔUA**2S**-Glc**NS6S**-IdoA**2S**-Glc**NS2S** (90%)^a^	76 (2)	3	61 (6)	3
Ron G11237	ΔUA**2S**-Glc**NS6S**-IdoA**2S**-Glc**NS2S** (37%)	81	1	ND	1
	ΔUA**2S**-Glc**NS6S**-IdoA**2S**-An**S**1,6an (18%)				
	ΔUA**2S**-Glc**NS6S**-IdoA**2S**-Man**NS6S** (18%)				
	ΔUA**2S**-Glc**NS6S**-GlcA-Glc**NS3S6S** (8%)				
	ΔUA**2S**-Glc**NS6S**-GlcA-Glc**NS6S** (12%)^b^				
SAGAG Peak 1	ΔUA**2S**-Glc**NS6S** + ΔUA**2S**-Glc**NS**^c^	46	1	55	1
SAGAG Peak 2	ΔUA**2S**-Glc**NS6S** + ΔUA-Glc**NS6S**^c^	28	1	47	1
SAGAG Peak 3	ΔUA**2S**-Glc**NS6S** + ΔUA**2S**-Glc**NS6S**^c^	68	1	66	1
**SYNTHETIC TETRASACCHARIDES**					
T7	IdoA-GlcNAc**6S**-IdoA-GlcNAc**6S**-(CH_2_)_5_NH_2_	0	1	0 (0)	3
T11	IdoA-GlcNAc**6S**-IdoA**2S**-GlcNAc**6S**-(CH_2_)_5_NH_2_	0	1	0 (0)	3
T13	IdoA-Glc**NS6S**-IdoA-Glc**NS6S**-(CH_2_)_5_NH_2_	0	1	0 (0)	3
T38	IdoA-Glc**NS6S**-IdoA**2S**-Glc**NS6S**-(CH_2_)_5_NH_2_	24	1	10 (10)	3
T39	IdoA**2S**-GlcNAc**6S**-IdoA**2S**-GlcNAc**6S**-(CH_2_)_5_NH_2_	0	1	0 (0)	3
T40	IdoA**2S**-Glc**NS6S**-IdoA**2S**-Glc**NS6S**-(CH_2_)_5_NH_2_	30	1	16 (3)	3

*Values are expressed as percentage inhibition compared to control values without inhibitor expressed as mean ± SD or from a single experiment. GAGs were added in a concentration of 100 ug/ml in the LP and C4 assay*.

a *Composition and purity by NMR spectroscopy*.

b *Composition by NMR spectroscopy*.

c *Composition after digestion of the tetrasaccharide with a cocktail of heparinase I, II, and III followed by RPIP-HPLC disaccharide identification*.

### MBL-MASP Complex Binds to Immobilized Heparin and Tissue Heparan Sulfate

**Figure 5:**
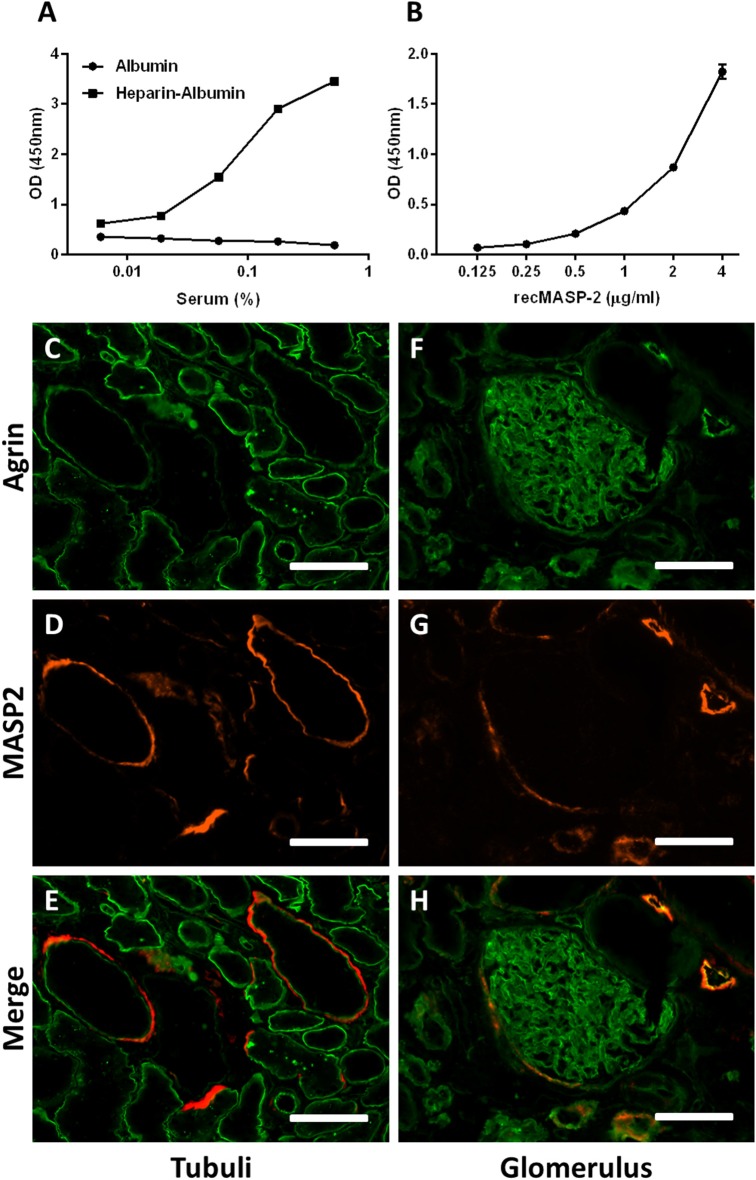
HSPG's might serve as a docking platform for the MBL/MASP complex. **(A)** Incubation of serum on a heparin-albumin coated plate results in binding of MASP-2 to the heparin. **(B)** recMASP-2 binds to heparin-albumin coated on a plate. **(C–H)** Confocal immunofluorescence double staining of human donor kidneys for MASP-2 (red) and agrin (green) showed partial co localization in the basement membranes of some tubuli, within Bowman's capsule and some blood/lymph vessel basement membranes. Data is expressed as representative measurement **(A)** or as mean ± SEM of two independent experiments **(B)**. Scale bars indicate 50 μm.

We have shown in this study that specific domains in heparins and HS can inhibit the LP of complement by inhibiting the enzymatic activity of the MASP2 enzyme. To test whether the MASP-2 in serum indeed binds to solid phase heparin/HS, we incubated serum in heparin-albumin coated wells and measured whether MASP-2 was bound to the immobilized heparin. Results showed binding of serum-derived MASP-2 to immobilized heparin in a dose dependent fashion (Figure 5A), indicating that serum MASP-2 could bind to HS/heparin on cells and tissues. RecMASP-2 showed binding to heparin albumin as well (Figure 5B).

**Figure 6:**
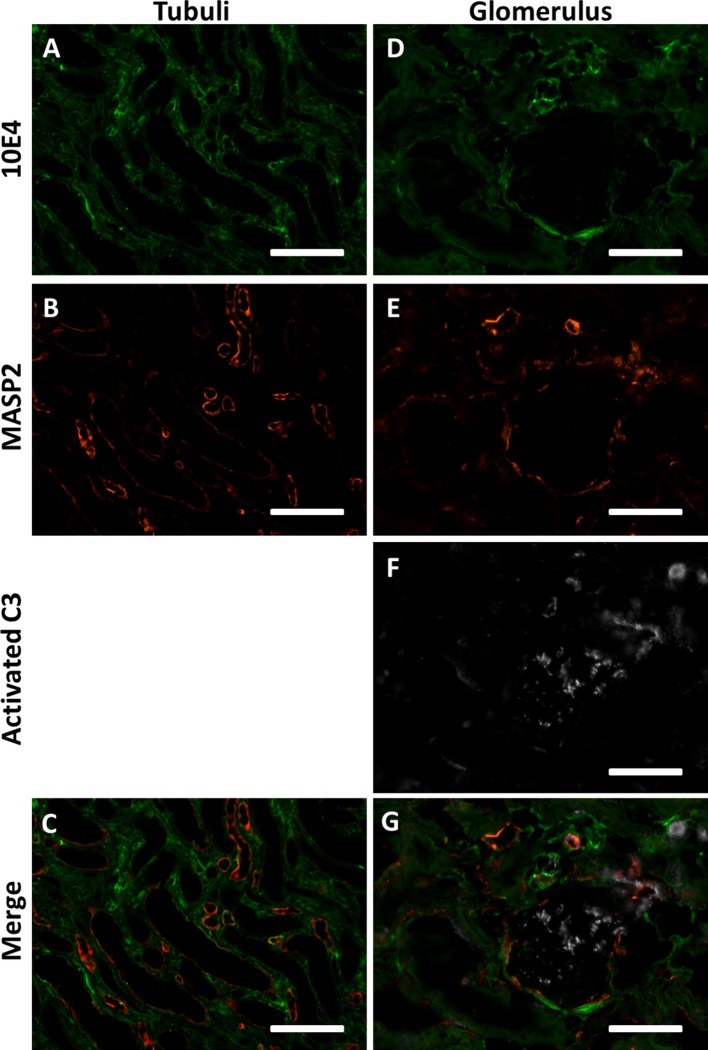
MASP2 is bound to HSPGs in an enzymatically inactive state. **(A–C)** Confocal immunofluorescence double staining of HS marker 10E4 (green) showed partial co localization with MASP-2 (red) in some tubular and vascular basement membranes of human donor kidneys. **(D–G)** Confocal immunofluorescence triple staining for HS mAb 10E4 (green), MASP-2 (red) and cleaved C3 (white). MASP-2 colocalized partially with HS in some vascular basement membranes and Bowmans capsule, but not with activated C3. Activated C3 is predominantly localized in the glomerulus (single positive) and not seen where MASP-2 co localizes with 10E4, indicating that MASP2 is bound the HSPGs in an inactive state. Scale bars indicate 50 μm.

We thus hypothesized that HS in extracellular matrices and cell membranes could function as docking platforms for MASP-2 *in vivo*. To test this hypothesis we double stained human donor kidneys for MASP-2 and basement membrane HS proteoglycan agrin, which is localized in renal glomerular, tubular and vascular basement membranes. We also performed a double staining for MASP-2 and HS (mAb 10E4). Both double stainings revealed MASP-2 to be present in a subset of tubular basement membranes, partly colocalizing with agrin and HS (Figures 5C–H, 6A–C). The fact that not all basement membranes showed MASP-2 positivity might be because MASP-2 is just very locally produced and stored in the underneath basement membrane, and/or that just a small subset of renal basement membranes displayed HS with a MASP-2 binding motif.

However, from these data it is not clear whether MASP-2 is enzymatically active or not when co-localized to basement membrane HSPGs. Therefore, we performed a triple staining for HS using mAb 10E4, MASP-2 and C3b, the cleaved product of C3, also called active C3. Although the 10E4 and MASP-2 epitopes of HS are different from each other, in Figures 6D–G it can be appreciated that MASP-2 and HS showed partial co localization in some basement membranes of vascular structures and in Bowman's capsule. Cleaved C3 within the glomerulus did not colocalize with MASP-2 and/or HS. It can also be seen that cleaved C3 occasionally showed co localization with MASP-2, but not when MASP-2 is co localized with 10E4/HS. Within the limitations of this approach, we speculate that MASP-2 bound to tissue HS is not able to activate the LP and thus no C3b can be formed i.e., MASP-2 might be enzymatically inhibited by the binding to HSPGs.

## Discussion

The interaction of GAGs (especially heparin-related GAGs) with complement has been known for some decades, but it has never been tested on a larger scale whether GAGs and derivatives thereof could be specific inhibitors of either of the complement pathways. In this study we tested a library of GAG-related polysaccharides for their complement inhibitory capacity and showed that the LP of complement can be specifically blocked by some HS and heparin- and HS-derived oligosaccharides. Decreasing the disaccharide length of the HS and heparin oligosaccharides resulted in more specificity for the LP and loss of CP and AP inhibitory activity. Since the LP shares the C3 convertase C4b2a with the CP, LP-specific GAGs must inhibit either the MASP enzymes or the pattern recognition molecules of the LP. Our results show that heparin fragments inhibited the cleaving of C4, but did not affect the binding of MBL to mannan, suggesting a blocking effect of GAGs on either MASP-1 or MASP-2 activity. This conclusion is further strengthened by the finding that the tested GAGs are also able to inhibit the LP initiated by ficolin-3 as a pattern recognition molecule. We could also show binding of serum-derived MASP-2 as well as recMASP-2 to immobilized heparin and demonstrated the binding affinity of recombinant MASP-2 to heparins and HS. Moreover, partial co-localization was seen of MASP-2 and agrin/10E4 HS epitope in some basement membrane of tubuli and vessels in human kidney, while activated C3 did not co-localize. These data might suggest that HS *in situ* might function as a docking platform for MASP-2 rendering the enzyme inactive, thereby eventually protecting the tissue from LP complement activation.

GAG length, disaccharide backbone composition, and sulfation pattern form the main determinants for protein binding properties of GAGs in general and this study shows that this holds true for complement components as well. Although the heparin/HS (oligosaccharides) were tested in a fixed μg/ml concentration, increasing the difficulty to directly compare small and large fractions, it can be appreciated that in larger GAGs, the complement inhibitory capacity is predominantly determined by sulfation degree and less by disaccharide composition and linkages. GAGs carrying a different backbone compared to heparin like O-sulfated *E. coli* K5 polysaccharide, and GAG-like polysaccharides fucoidan and dextran sulfate show equally strong inhibition of all complement pathways, stressing the role of GAG sulfation in complement inhibition. It can be appreciated from Table 2 that in most cases the inhibitory capacity of GAGs is comparable for the CP and AP. This might suggest that the CP and AP share an inhibitory GAG binding protein and that the GAG binding site in the LP is unique within the complement system. The known interactions between GAGs and the complement system have been summarized by us before (47) and we have more extensively investigated the interaction between GAGs and properdin (12). Interestingly, the GAGs showing the highest affinity for properdin in the study conducted earlier also show the strongest CP and AP inhibition (12). This might indicate a role for the GAG/properdin interaction in our current results. Properdin is a stimulator of the AP, by stabilizing the C3 convertase, but can have the same effect in the CP since cleaving of C3 by the C3 convertase C4bC2a can be followed by AP activation. Whether the inhibition by GAGs of the CP and AP is indeed properdin mediated or not should be studied further, since interactions between GAGs on one hand and AP activator properdin, AP inhibitor factor H (13) and CP inhibitors like C1-inhibitor on the other hand have been described Caughman et al. (48).

This study showed that smaller heparin and HS depolymerization products lose their ability to inhibit the CP and AP and become specific inhibitors for the LP. HS oligosaccharides start to be LP specific from dp12 and heparin from dp6. Our surface plasmon resonance experiments indicated that MASP-2 recognizes a small GAG epitope. Of note, heparin is significantly more sulfated than HS (~2.5 and ~0.85 sulfates per disaccharide, respectively). Furthermore, HS oligosaccharides were generated using heparinase III, which specificity for (GlcNAc ± 6S–GlcA) disaccharides will produce fragments with low/non-sulfated saccharide extremities. We therefore assume that larger (dp6–dp12) HS oligosaccharides may contain just one MASP2 binding site connected to low sulfated, biologically inactive HS stretches. Whereas, larger (dp6–dp12) heparin oligo's, due to high sulfation, also start to recognize C1inh, ATIII and other members of the complement serine proteases and therefore start to inhibit the CP and AP besides the LP. The smallest component capable of inhibiting the LP is a tetrasaccharide, which needs to have at least pentasulfation to show inhibitory function. This is interesting because other GAG/protein interactions e.g., heparin and ATIII have shown to require larger high sulfation stretches. Therefore, the interaction of a tetrasaccharide with MASP enzymes holds clinical potential. Interestingly, replacing the iduronic acid at the non-reducing end of the tetrasaccharide by unsaturated ΔHexA could increase the inhibitory potential, suggesting the importance of the backbone structure. Alternatively, the reducing terminal—(CH_2_)_5_NH_2_ derivatization of the synthetic tetrasaccharides might interfere the interaction with MASP-2 to some extent.

**Figure 7:**
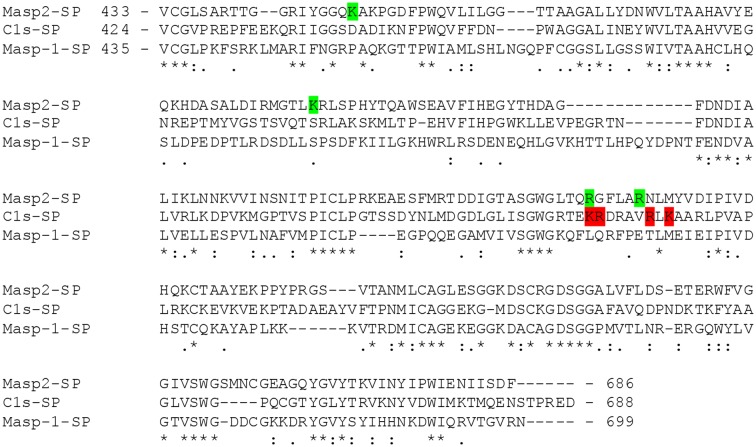
Amino acid sequence alignment of the serine protease domains of MASP-2, C1s and MASP-1. Sequence alignment was done by Clustal W (https://www.genome.jp/tools-bin/clustalw) as described by Thompson et al. (53). Amino acid sequences were obtained in Uniprot (https://www.uniprot.org/). Indicated in green are the positively charged arginine and lysine residues in MASP-2 involved in the recognition of the tyrosine-sulfate residues in C4 (K450, K503, R578, and R583). In red these residues are shown in C1s (K575, R576, R581, and K583). As can be seen, the positively charged exocites of MASP-2 and C1s clearly differs from each other, while such a positively charged exosite is lacking in MASP-1.

We showed in this study that the inhibitory effect of GAGs on the lectin route of complement is not pattern recognition molecule related, but rather MASP related. As elegantly shown by Héja and colleagues, MASP-2 is solely responsible for the activation of C4 and both MASP-1 and 2 are responsible for the activation of C2 (46). This study shows that GAGs inhibit the C4 activation and that GAGs show interaction with the catalytic domain of MASP-2. Therefore, we can conclude that GAGs interact with MASP-2 and inhibit the enzymatic activity. Literature has demonstrated that MASP-2, but not MASP-1, has a highly positively charged exosite located in the serine protease domain, which differs from de described exosite in C1s (49, 50). Under physiological conditions, this positively charged exosite is believed to be the binding site of C4. C4 carries a negatively charged cluster consisting of three tyrosine-sulfate residues, interaction of these tyrosine-sulfate residues with the positively charged exosite on MASP-2 and C1s is crucial for cleaving, and therefore activity, of C4 (51, 52). Since heparin, and HS to a lesser extent, is negatively charged due to their sulfate groups, we propose that specific LP inhibition by HS and heparin oligosaccharides occurs *via* binding to this positively charged exosite. In Figure 7, we aligned the amino acid sequence of the serine protease domains of MASP-2, MASP-1, and C1s. In green we indicated the positively charged arginine and lysine residues in MASP-2 involved in the recognition of the tyrosine-sulfate residues in C4 (K450, K503, R578, and R583). In red we showed them in C1s (K575, R576, R581, and K583). As can be seen, such an exosite is lacking in MASP-1. Moreover, MASP-1 is not cleaving C4, just C2. Moreover, because we found the inhibition of the C4 deposition assay we studied MASP-2 and not MASP-1. Therefore, these data strongly suggest that the heparin oligo's block the interaction of MASP-2/C4 interaction at the interface of the MASP-2 exosite with the tyrosin-sulfate residues of C4, and thereby prevents cleavage of C4.

It has been described that protease inhibitors like C1inh, ATIII, and tissue factor pathway inhibitor can form complexes with the MASP proteases (15, 54) and could therefore influence our results by heparin mediated activation of these serine protease inhibitors followed by inactivation of the MASP enzymes by proteolytic cleavage. We deem it however unlikely since ATIII needs minimally a 3-O sulfated pentasaccharide which is not present in the synthetic tetrasaccharides tested (25). C1inh and TFPI are also not very likely involved, since according to literature both protease inhibitors show an increased protease activity at low heparin concentrations and blocking of the protease activity at high heparin concentrations (15, 55). We never observed such kinetics using the WIELISA or C4 deposition assays. Besides, the 1M NaCl conditions in the C4 deposition assay the MASPs will not react with the serpins.

This study shows that the serum-derived MASP-2 can bind to heparin and HS GAGs and inhibit the LP of complement. These findings suggest that HS proteoglycans *in vivo* can act as a docking station for MASP-2 and preclude LP activity. In support to this, we found some co-localization of MASP-2 and HS proteoglycan agrin and HS in Bowman's capsule and the basement membranes of some tubuli and vessels. We were not able to show cleaved C3 to co-localize with HS and MASP-2. To test HS-dependent MASP-2 binding renal sections were pretreated with heparitinase. Effective enzymatic cleavage of HS was shown by induced staining using anti-ΔHS stub mAb 3G10, which after heparitinase became brilliant positive in all extracellular matrices of the kidney. Nevertheless, the intensity of the MASP-2 staining did not diminish after heparitinase (data not shown). This could mean that MASP-2 is not physically bound with HS. However, we cannot exclude that its binding to HS precludes the heparitinase enzyme to cleave HS. Another explanation could be that the MASP-2 in renal tissue is part of a larger complement complex, including MASP-1, MBL, and eventually other complement factors (C2 and C4 eg). These other complement factors also might interact with renal determinants, other than HS. This means that digestion of HS will not reduce MASP-2 presence. All in all, such an enzymatic approach is not conclusive. Although our immunofluorescence data is far from robust proof, this might suggest that MASP-2 is stored inactively in the basement membrane and we speculate that it might be released upon the right stimuli e.g., by the action of heparanase or oxidative stress.

We show in this study that HS and small heparin fragments are specific LP inhibitors, although the interaction is in the micromolar affinity range resulting in rather high IC50 values. To date there is no specific LP inhibitor clinically available, although a phase II clinical trial in renal patients using humanized anti-MASP-2 mAb (OMS721 by OMEROS, Saettle WA, USA) was recently closed with promising results in patients suffering from IgA nephropathy and lupus nephritis. Furthermore, an interesting study was recently published introducing a modified TFPI 1 domain as a potential MASP-2 inhibitor (56). Current treatment option for LP mediated conditions would be C5 inhibitor eculizumab. However, eculizumab is very expensive and inhibits all three complement pathways. The use of polysaccharide-based therapeutics has the advantages: production can be cheap and easy, and polysaccharides are not susceptible to proteolytic enzymes, making them less vulnerable to degradation. Moreover, decades of experience has been obtained with heparin based therapeutics in the field of anti-coagulation. Therefore, we believe that polysaccharide based specific LP inhibitors have good potential for further development. The comparison of four different β-elimination heparin-derived tetrasaccharide fragments and synthetic tetrasaccharides revealed the best LP/MASP inhibition by the fully hexasulfated ΔHexA, 2S-GlcNS, 6S-ΔHexA,2S-GlcNS, 6S structure. This structure could be a promising starting molecule to develop related small glycan-based LP/MASP inhibitors with higher affinity.

## Data Availability Statement

All datasets generated for this study are included in the article/Supplementary Material.

## Author Contributions

DT, MD, CS, MS, SB, and JB contributed to the conception and design of the study. DT, FP, WD, AM-A, and RV conducted the experiments. AN, PC, G-JB, and PG delivered crucial reagents. DT wrote the first draft of the manuscript. All authors contributed to manuscript revision, read, and approved the submitted version.

## Conflict of Interest

The authors declare that the research was conducted in the absence of any commercial or financial relationships that could be construed as a potential conflict of interest.
