# Supplementary figures and images for: MASP-2 Is a Heparin-Binding Protease; Identification of Blocking Oligosaccharides
Source: Front Immunol. 2020 Apr 28;11:732. doi: 10.3389/fimmu.2020.00732 (PMC7212410; doi:10.3389/fimmu.2020.00732)

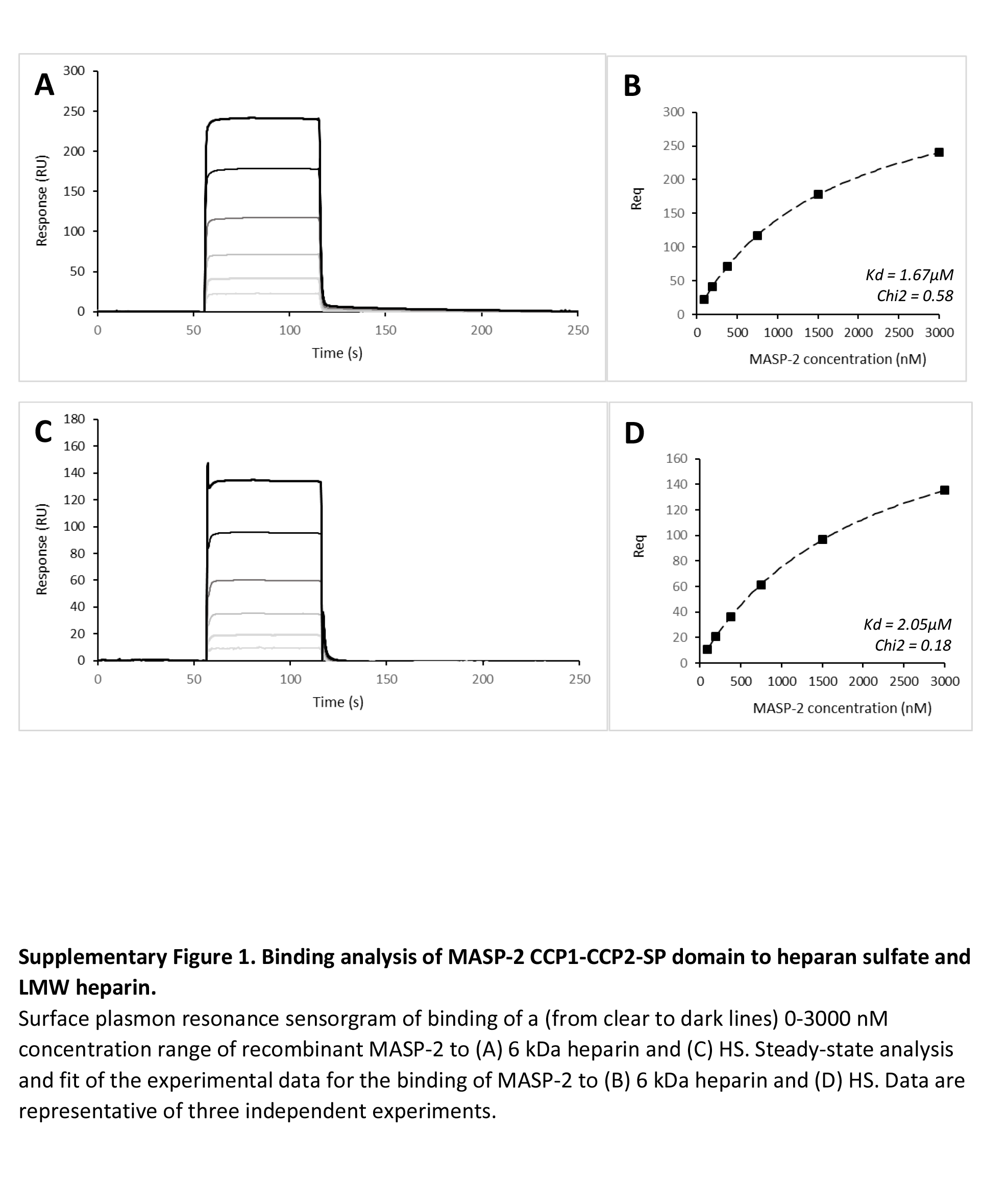

Supplement: Supplementary file 1 [file Image_1.TIF]
